# Supplementary material for: A Drosophila Su(H) model of Adams-Oliver Syndrome reveals cofactor titration as a mechanism underlying developmental defects
Source: PLoS Genet. 2022 Aug 11;18(8):e1010335. doi: 10.1371/journal.pgen.1010335 (PMC9398005; doi:10.1371/journal.pgen.1010335)
Supplement: S1 Table — (DOCX) [file pgen.1010335.s001.docx]

**S1 Table**: Calorimetric Binding Data for Native and Variant RBPJ/Su(H) Proteins

|  | | | | |  | |  | |  | |  | |
| --- | --- | --- | --- | --- | --- | --- | --- | --- | --- | --- | --- | --- |
| **Ligand** | **Macromolecule** | **ΔG° (kcal/mol)** | **ΔH° (kcal/mol)** | **ΔS (kcal/mol(K))** | | **TΔS° (kcal/mol)** | | **K (M^-1^)** | | **K_D_ (nM)** | |  |
| DNA | Su(H) WT | -8.7 ± 0.1 | 8.5 ± 0.2 | 60.8 ± 0.3 | | 17.2 ± 0.1 | | 5.4 ± 0.8 x 10^6^ | | 188.5 ± 27.4 | |  |
|  | Su(H) E137V | -7.9 ± 0.1 | 10.1 ± 0.5 | 63.4 ± 1.7 | | 18.0 ± 0.5 | | 1.2 ± 0.1 x 10^6^ | | 841.6 ± 98.2 | |  |
|  | Su(H) K132M | -8.0 ± 0.1 | 11.2 ± 0.4 | 67.7 ± 1.2 | | 19.2 ± 0.3 | | 1.5 ± 0.1 x 10^6^ | | 689.9 ± 48.7 | |  |
| DNA | RBPJ WT | -9.2 ± 0.1 | 7.8 ± 0.1 | 60.1 ± 0.0 | | 17.0 ± 0.0 | | 1.4 ± 0.2 x 10^7^ | | 74.0 ± 10.8 | |  |
|  | RBPJ E89G | -8.2 ± 0.1 | 12.0 ± 0.8 | 71.5 ± 2.5 | | 20.2 ± 0.7 | | 2.4 ± 0.5 x 10^6^ | | 440.5 ± 86.8 | |  |
|  | RBPJ K195E | -7.7 ± 0.1 | 5.7 ± 0.9 | 47.3 ± 2.9 | | 13.4 ± 0.8 | | 9.0 ± 2.0 x 10^5^ | | 1163.7 ± 258.1 | |  |
| dNotch RAM | Su(H) WT | -9.2 ± 0.0 | -13.2 ± 0.1 | -13.5 ± 0.3 | | -4.0 ± 0.1 | | 5.4 ± 0.2 x 10^6^ | | 186.7 ± 8.3 | |  |
|  | Su(H) E137V | -9.3 ± 0.2 | -13.3 ± 0.7 | -13.6 ± 2.8 | | -4.1 ± 0.8 | | 6.5 ± 1.7 x 10^6^ | | 166.3 ± 47.4 | |  |
|  | Su(H) K132M | -9.6 ± 0.3 | -16.2 ± 3.5 | -22.1 ± 12.4 | | -6.6 ± 3.7 | | 1.2 ± 0.6 x 10^7^ | | 106.7 ± 61.3 | |  |
| mNotch1 RAM | RBPJ WT | -10.5 ± 0.0 | -12.5 ± 0.2 | -6.9 ± 0.6 | | -2.1 ± 0.2 | | 4.8 ± 0.3 x 10^7^ | | 20.8 ± 1.2 | |  |
|  | RBPJ E89G | -10.6 ± 0.2 | -12.1 ± 0.1 | -5.2 ± 0.9 | | -1.5 ± 0.3 | | 5.6 ± 1.6 x 10^7^ | | 19.9 ± 7.0 | |  |
|  | RBPJ K195E | -10.5 ± 0.2 | -12.2 ± 0.3 | -6.0 ± 1.4 | | -1.8 ± 0.4 | | 5.1 ± 1.8 x 10^7^ | | 22.4 ± 7.8 | |  |
| Hairless | Su(H) WT | -11.7 ± 0.1 | -16.2 ± 1.3 | -15.1 ± 4.7 | | -4.5 ± 1.4 | | 3.6 ± 0.4 x 10^8^ | | 2.8 ± 0.3 | |  |
|  | Su(H) E137V | -12.1 ± 0.5 | -16.1 ± 1.4 | -13.5 ± 6.3 | | -4.0 ± 1.9 | | 1.1 ± 0.9 x 10^9^ | | 1.7 ± 1.0 | |  |
|  | Su(H) K132M | -11.8 ± 0.8 | -16.5 ± 0.2 | -15.8 ± 2.7 | | -4.72 ± 0.8 | | 7.9 ± 5.9 x 10^8^ | | 5.1 ± 5.9 | |  |
| SHARP | RBPJ WT | -12.0 ± 0.9 | -11.7 ± 0.5 | 1.1 ± 1.3 | | 0.3 ± 0.4 | | 1.5 ± 1.7 x 10^9^ | | 3.5 ± 3.3 | |  |
|  | RBPJ E89G | -11. ± 0.4 | -10.5 ± 1.4 | 3.0 ± 5.8 | | 0.9 ± 1.7 | | 2.9 ± 1.8 x 10^8^ | | 5.2 ± 3.0 | |  |
|  | RBPJ K195E | -10.5 ± 0.3 | -13.3 ± 0.9 | -9.3 ± 3.8 | | -2.8 ± 1.1 | | 5.8 ± 2.4 x 10^7^ | | 21.1 ± 10.1 | |  |
