# Supplementary figures and images for: A Drosophila Su(H) model of Adams-Oliver Syndrome reveals cofactor titration as a mechanism underlying developmental defects
Source: PLoS Genet. 2022 Aug 11;18(8):e1010335. doi: 10.1371/journal.pgen.1010335 (PMC9398005; doi:10.1371/journal.pgen.1010335)

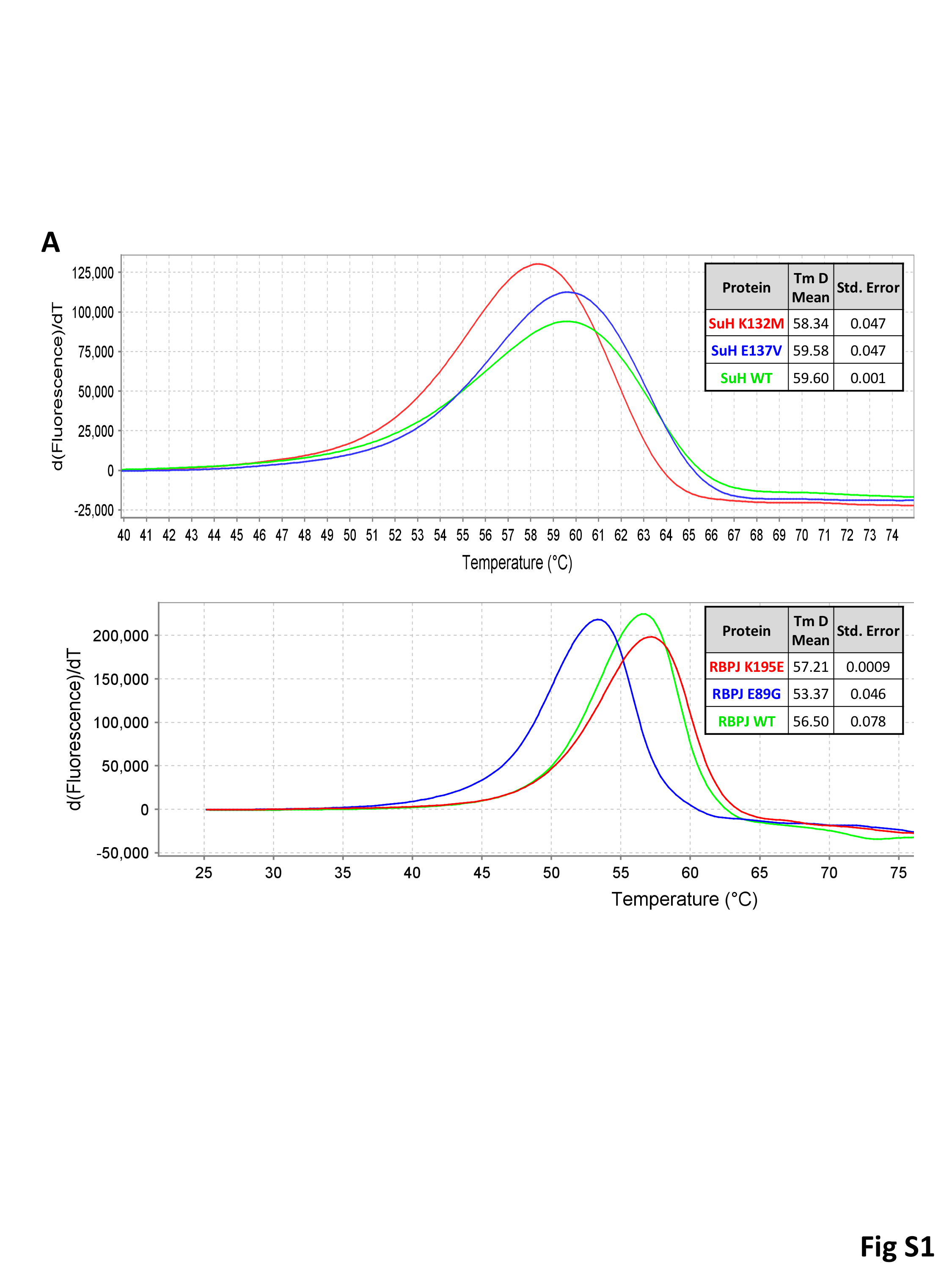

Supplement: S1 Fig — The mean melting temperature (Tm) and standard error were calculated from triplicate experiments. (TIFF) [file pgen.1010335.s002.tiff]

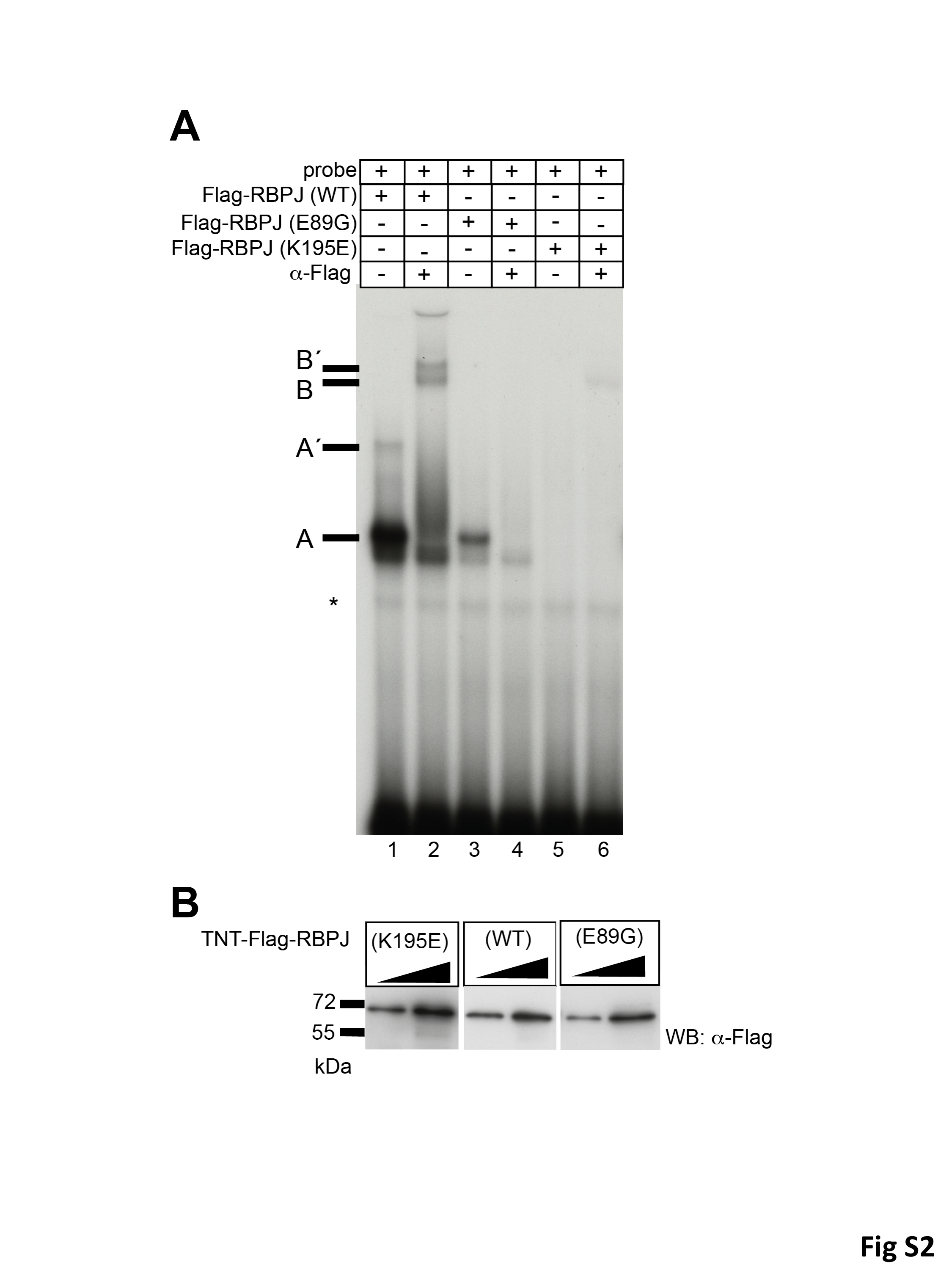

Supplement: S2 Fig — Full length Flag-Rbpj WT (lane 1 and 2) binds to DNA in contrast to Flag-Rbpj K195E mutant (lane 5 and 6) and Flag-Rbpj E89G mutant (lane 3 and 4), which interact weakly with DNA. Protein-DNA interaction of Flag-Rbpj WT is shown by complex A (single occupancy, lane 1 and 2), complex A’ (double occupancy, lane 1 and 2) and complex B and B’ with the addition of anti-Flag showing supershifting (lane 2). Bottom panel: Western blot to show similar expression levels of WT and variant Rbpj constructs. (TIFF) [file pgen.1010335.s003.tiff]

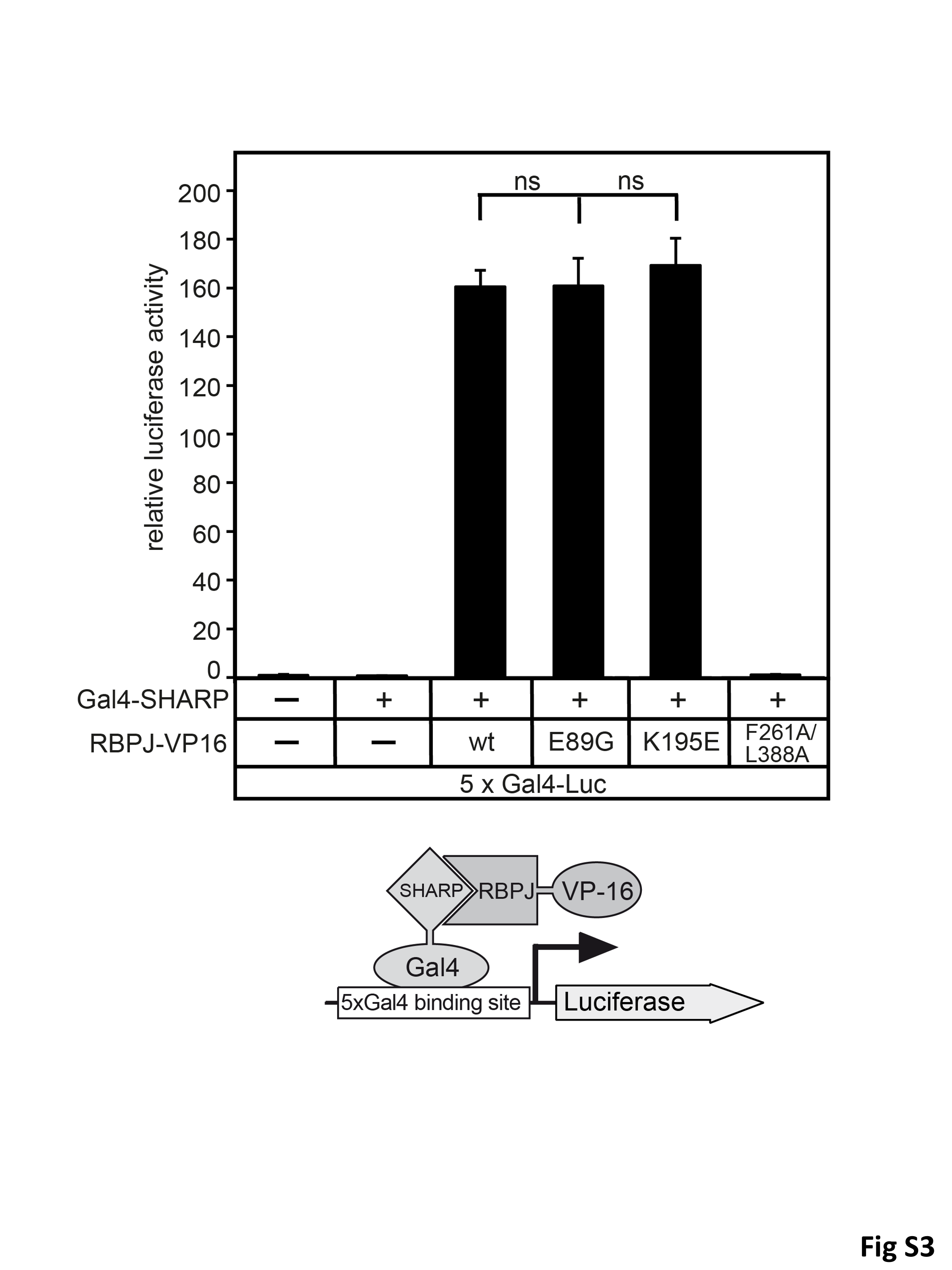

Supplement: S3 Fig — HeLa cells were cotransfected with the indicated Gal4-SHARP (300ng) and Rbpj-VP16 (50ng) constructs together with the pFR-Luc reporter (500ng), which contains five Gal4 DNA binding sites upstream of the luciferase gene. Relative luciferase activity was determined after cotransfection of the pFR-Luc reporter construct alone. WT, E89G, and K195E activate the reporter similarly, suggesting that the AOS variants bind SHARP similar to WT Rbpj, whereas the Rbpj double-mutant F261A/L388A, which is compromised for SHARP binding, does not activate the reporter. (TIFF) [file pgen.1010335.s004.tiff]

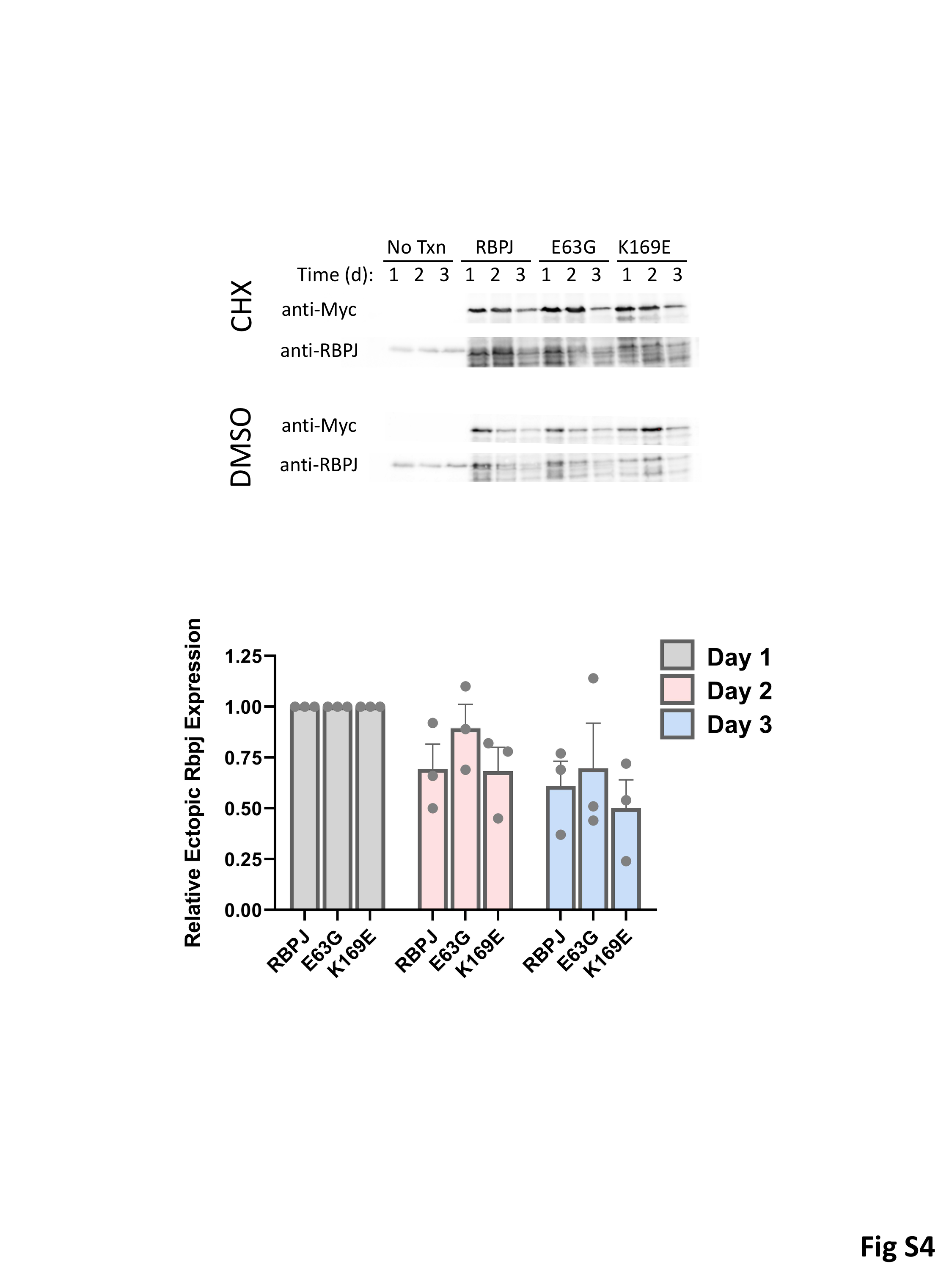

Supplement: S4 Fig — Ectopic expression of Myc-tagged RBPJ constructs was detected by western blot analysis with anti-Myc (9B11) and anti-RBPJ antibodies. The signal intensities of corresponding protein bands were quantified by ImageJ software and expressed as ratios relative to those at day 1. (TIFF) [file pgen.1010335.s005.tiff]

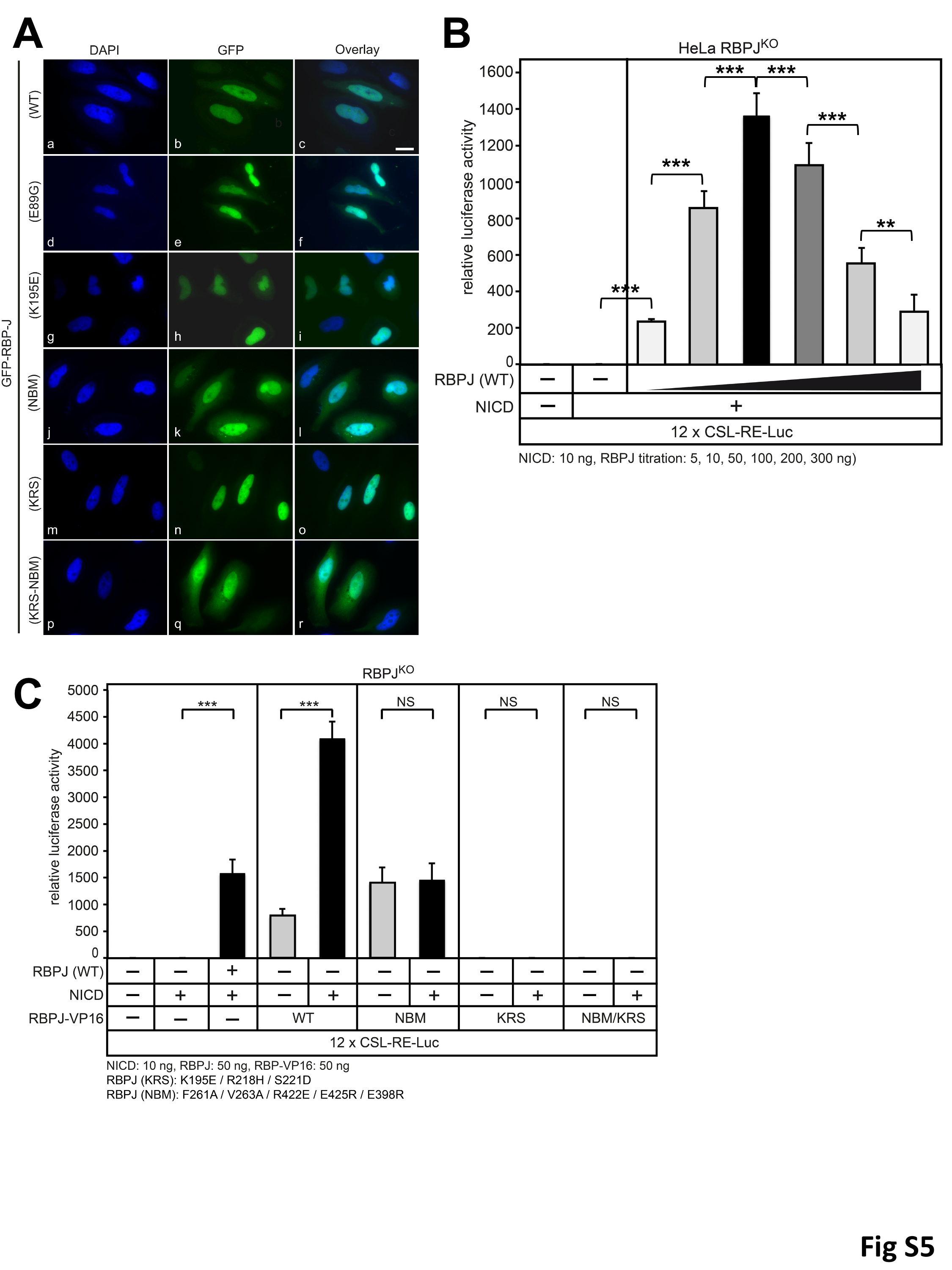

Supplement: S5 Fig — A. Subcellular localization of Rbpj proteins after expression in HeLa cells, illustrating predominantly nuclear localization of both WT and variant Rbpj proteins. HeLa cells were transiently transfected with 0.3 μg of the respective GFP-Rbpj plasmids: WT, E89G, K195E, NBM (NICD binding mutant: F261A/V263A/R422E/E425R/E398R), KRS (DNA binding mutant: K195E/R218H/S221D) or NBM/KRS. After 24 hours, cells were fixed and stained with DAPI and imaged under a fluorescent microscope using a 63x objective. B. Luciferase reporter assays using different levels of WT Rbpj expression. HeLaRBPJ-KO cells were co-transfected with the Notch/RBPJ dependent reporter 12xCSL-RE-Luc (250 ng), without or with NICD (10 ng), and Rbpj (5, 10, 50, 100, 200, 300 ng). Luciferase activity was measured 24 hours after transfection. Maximal reporter activity was observed with 50 ng of transfected Rbpj DNA. Bars represent mean values from six independent experiments and error bars indicate standard deviation. ([**] P < 0.001, [***] P < 0.0001, unpaired Student’s t-test). C. Luciferase reporter assays demonstrating that Rbpj mutants NBM and KRS are unable to bind NICD and DNA, respectively. HeLaRBPJ-KO cells were transfected with the Notch/RBPJ dependent reporter 12xCSL-RE-Luc (250 ng) without or with the indicated Rbpj or Rbpj-VP16 constructs (50 ng), and with or without NICD (10 ng). Luciferase activity was measured 24 hours after transfection. While Rbpj-VP16 NBM is able to activate the reporter, addition of NICD does not result in an addition increase in reporter activity due to Rbpj NBM unable to bind NICD. Rbpj-VP16 KRS and NBM/KRS are unable to activate the reporter with or without NICD. Bars represent mean values from six independent experiments and error bars indicate standard deviation. ([***] P < 0.0001, [NS] not significant, unpaired Student’s t-test). (TIFF) [file pgen.1010335.s006.tiff]
