# Supplementary material for: A Drosophila Su(H) model of Adams-Oliver Syndrome reveals cofactor titration as a mechanism underlying developmental defects
Source: PLoS Genet. 2022 Aug 11;18(8):e1010335. doi: 10.1371/journal.pgen.1010335 (PMC9398005; doi:10.1371/journal.pgen.1010335)
Supplement: Attachment — Submitted filename: Rebuttal_PLOS_Genetics_Final.docx [file pgen.1010335.s007.docx]

We would like to thank the reviewers for their knowledgeable and insightful comments. Overall, we were very encouraged by the positive feedback we received from the three reviewers, which included comments such as, Reviewer1 “*This is very nice paper. The data are clear and convincing*”; Reviewer2 “*This is a well-constructed study that serves to connect previous observations from flies and patients in a clear way*”; and Reviewer3 “*The experiments are well performed and described and convincingly support the model proposed*.” We also welcome the opportunity to address the reviewers’ comments in our revised submission. As summarized point-by-point below, we thoughtfully address all of the issues raised by the reviewers, which in our opinion results in an improved manuscript worthy of publication in PLOS Genetics.

Reviewer1:

1. *it would be nice to show that the T4 and O5 alleles indeed behave as loss-of-function alleles in the absence of wild-type Su(H) (see point 2 below).*

For clarity, we combined our response to this point with point #2 below.

2. *two other gof alleles of Su(H) have been described. The eBC11 mutation (Y134N, see Flybase) maps very close to the O5 and T4 mutations studied here and might therefore affect DNA-binding. The S5 mutation (R266H) was previously shown to impair DNA-binding. Therefore, the main conclusion of the current study is likely to apply to these two alleles. This could be discussed.
   In further support of this piece of work, the T4 and S5 alleles were previously suggested to behave as loss-of-function alleles when homozygous in mosaic genetics. This observation led the authors to suggest that these mutant proteins may act by titrating Hairless. This could also be discussed.*

We thank the reviewer for raising these two points about prior studies using the T4 and O5 alleles as well as the eBC11 allele and S5 mutation. We agree this was an omission on our part and we have now added the following sentences highlighting the major findings from these prior genetic studies and integrate how they are consistent with our new molecular data within the Discussion of the paper:

*“Our findings support the idea that the Su(H)^T4^ (E137V) and Su(H)^O5^ (K132M) variants act as dominant negative proteins due to sequestering cofactors, especially in sensitized backgrounds where either Notch or Hairless are limiting. It should be noted that a similar mechanism was proposed and demonstrated by over-expressing a DNA binding deficient Rbpj (R218H) molecule in mammalian cell culture^42^, which is also consistent with our cellular reporter assays using the Rbpj DNA binding mutants KRS and K195E (****Fig 7E****). This mechanism also likely explains the previously observed dominant gain-of-function activity of other described Su(H) alleles, such as Su(H)^eBC11^ (Y134N)^17,43^ and Su(H)^S5^ (R266H)^26,37^. While the impact of the Su(H)^eBC11^ Y134N variant on DNA and cofactor binding has yet to be experimentally tested, its location within the DNA binding domain and the reported dominant genetic behavior of the Su(H)^eBC11^ allele support the idea this variant is also likely to selectively compromise DNA binding. Moreover, the Su(H)^S5^ R266H variant has been previously proposed to have dominant negative activity based on prior studies showing that it qualitatively disrupts DNA binding and was found to still interact with the Hairless protein in a yeast-two hybrid assay^44^. Our quantitative molecular and genetic studies further support these ideas and also help to explain how homozygous mutant clones of the Su(H)^T4^ allele cause a Notch loss-of-function phenotype^16^; namely, the loss of DNA binding by the Su(H)^T4^ variant will result in the failure to robustly bind key target genes downstream of Notch activation. Thus, the Su(H)^T4^ allele should be viewed as both a dominant negative allele that can interfere with wild type Su(H) function in heterozygous cells by binding limiting co-factors, and a hypomorphic loss-of-function allele due to decreased DNA binding and the subsequent failure to activate key Notch target genes in homozygous mutant cells.”*

3. *Typo (p 11): (compare H1/+ data in Fig 5B with the H1/S(H)-GFP data in Fig 5H).*

We thank Reviewer1 for pointing out this typo, which has been corrected in our revised manuscript.

Reviewer2:

1. *The authors have performed classic genetic interactions with Notch alleles and Hairless alleles that in essence recapitulate the findings from previously (e.g. Fortini et al, Ashburner) The data are much better explained and properly quantified here, but the findings are not novel, except for those showing the effects of increased Notch ( Notch-GFP BAC) that agree with the titration model. To make a step forward they could test other predictions from the model to make a more complete study. e.g. what do they predict from an extra copy of Hairless? for phenotypes from NICD expression? Or from combinations with an allele of Su(H) that has reduced Hairless binding?*

We thank the reviewer for these comments and the suggestion to further test how gene dose of the key activator (Notch), co-repressor (Hairless) and transcription factor (Su(H)) impact *Drosophila* phenotypes. As a complement to our studies in which we had previously added an extra copy of the *Notch* gene (Notch-GFP BAC), we performed a similar analysis by adding an extra copy of the Hairless gene using a previously published Hairless-GFP rescue transgene (Fig 3D). Intriguingly, the extra Hairless allele dramatically increased wing nicking phenotypes in the Su(H)T4 background, which further supports the idea that the impact of the DNA binding compromised Su(H) molecule is significantly enhanced when the levels of the Notch and Hairless co-factors are imbalanced. These data are now integrated with the other gene dose experiments and described in the following paragraph of the Results section of the paper:

*“These data show that flies heterozygous for the Su(H)^T4^ allele are susceptible to wing notching with a penetrance and severity that can be either enhanced in N^55e11^ heterozygotes (****Fig 3D****) or suppressed by adding an extra Notch allele (N-GFP in* ***Fig 3A****). Similarly, we found that the ability of the Su(H)^T4^ allele to induce wing nicking could also be suppressed by removing a Hairless allele (i.e. in Su(H)^T4/+^;H^1/+^ compound heterozygotes in* ***Fig 3A****). To further test how Hairless gene dose impacts the wing nicking phenotype induced by the Su(H)^T4^ allele, we used a previously published Hairless BAC rescue transgene (H-GFP)^19^ to add an extra Hairless allele in either wild type flies, Su(H)^T4/+^ heterozygotes, or Su(H)^IB115/+^ heterozygotes. Intriguingly, we found that flies with an extra Hairless allele developed normal wings in both a Su(H)^+/+^ wild type background and in Su(H)^IB115/+^ heterozygotes, whereas the extra H-GFP allele induced a dramatic increase in wing notching penetrance and severity in Su(H)^T4^ heterozygotes (****Fig 3D****). Taken together, these findings highlight how the gene dose of both Notch and Hairless can have a profound impact on the wing phenotypes generated by the Su(H)^T4^ allele.”*

In addition, we better highlighted both the novelty of our findings, and better described the non-intuitive results obtained by using the different Su(H) alleles in our discussion by adding the following paragraph: Note, this paragraph also addresses Points #3 and #4 below.

*“Third, our Drosophila genetic data generated by systematically testing the impact of both increasing and decreasing Notch and Hairless gene dose in the presence of different Su(H) alleles highlighted how genetic background can dramatically sensitize tissue-specific phenotypes. For example, flies heterozygous for the Su(H)^T4^ allele only have a weakly penetrant wing notching phenotype in a genetic background with two wild type alleles of Notch and Hairless. However, simultaneously lowering the Notch gene dose or increasing the Hairless gene dose dramatically enhanced the wing phenotype, whereas increasing Notch gene dose or decreasing Hairless gene dose suppressed the wing phenotype. Moreover, we similarly found that while the Su(H)^T4^ allele on its own did not dramatically impact macrochaetae development, simultaneously changing the relative dose of Notch and Hairless could significantly exacerbate macrochaetae phenotypes in compound heterozygotes with the Su(H)^T4^ allele. Collectively, these data suggest that genetic modifiers in the presence of the dominant-negative AOS-like Su(H) allele can have tissue-specific impacts based on whether they either strengthen or weaken the Notch co-activator complex versus Hairless co-repressor complex. Lastly, we also non-intuitively found that simply changing the gene dose of the Su(H) transcription factor can modify (i.e. partially suppress) each of these Notch- and Hairless-dependent phenotypes. Currently, we do not have a good molecular explanation for how decreasing the expression levels of the common transcription factor could suppress both Notch- and Hairless-dependent phenotypes, and future experiments are needed to define the complex relationships that exist between Notch, Hairless, and Su(H) gene dose in each specific tissue.”*

2. *Mammalian experiments do not really test the model, they validate the loss of DNA binding. Extending the mammalian cell experiments to show evidence in support of the titration model is needed.*

We agree with Reviewer2’s comments that the cellular assays in our original manuscript did not directly demonstrate that the titration model occurs in mammalian cells. However, in our revised manuscript (Fig 7E), we used luciferase reporter assays to provide evidence in support of the sequestration mechanism occurring in mammalian cells as well and added the following paragraph in the Results section.

*“Lastly, to test the sequestration mechanism in mammalian cells, we once again used our luciferase assays in HeLaRBPJ^-KO^ cells (****Fig 7E****). Because transfection of increasing amounts of WT Rbpj results in reduced reporter activity (****Fig 7E****), which has been reported previously^34,35^ and is likely due to titration of nuclear components, we designed three additional Rbpj mutants: (1) Rbpj^KRS^ (K195E/R218H/S221D), which combines the variant K195E with two other previously described Rbpj mutants that affect DNA binding^36,37^ and is completely devoid of any DNA binding (****Supplemental Fig 5****); (2) Rbpj^NBM^ (NICD Binding Mutant, F261A/V263A/R422E/E425R/E398R), which is unable to interact with NICD1 but retains its ability to bind DNA (****Fig 7E &*** ***Supplemental Fig 5****); and (3) Rbpj^NBM/KRS^, which is neither able to bind DNA nor NICD1. It should also be mentioned that Rbpj^NBM^ has been tested in Drosophila, using the fly ortholog Su(H), and in these in vivo assays was also shown to be completely lacking in NICD binding and Notch-mediated transcriptional activation^19^. For these experiments, a constant amount of WT Rbpj and NICD1 was co-transfected into cells to activate the reporter (****Fig 7E, panel i****), and increasing amounts of either WT Rbpj (****Fig 7E, panel ii****), Rbpj^NBM^ (****Fig 7E, panel iii****), Rbpj^KRS^ (****Fig 7E, panel iv****), Rbpj^NBM/KRS^ (****Fig 7E, panel v****), or the AOS variant K195E (****Fig 7E, panel vi****) were additionally transfected into the cells. These experiments demonstrate the following: (1) Increasing cellular concentrations of the Rbpj DNA binding mutants KRS and K195E both significantly reduce reporter activity more than WT Rbpj, albeit the reduction by the AOS variant K195E is modest compared to the KRS mutant; (2) Increasing cellular concentrations of Rbpj^NBM^, which cannot bind NICD1 but can still bind DNA, has only a very minor negative effect on reporter activity, likely by competing with WT Rbpj for DNA binding sites on the reporter; and (3) Increasing cellular concentrations of Rbpj^NBM/KRS^, which cannot interact with NICD1 or DNA, has no effect on reporter activity. Taken together, these data provide proof-of-principle, that Rbpj variants that are defective in DNA binding reduce Notch signaling via a cofactor sequestration mechanism.”*

3. *The wing nick phenotypes from [T4] and [O5] resemble Notch loss of function rather than Hairless, why do Notch loss of function effects predominate over those from titrating Hairless? Is this of relevance for the mammalian condition, can they demonstrate that there is titration of SHARP in mammalian cells?*

We thank the reviewer for this comment, which is a very interesting point. Our current thought is that each tissue, whether wing margin cells or macrochaetae, is differentially sensitive to specific components of the Notch pathway. For reasons we don’t fully understand, the Su(H)T4 allele is sufficient to compromise the Notch pathway and cause a weakly penetrant wing notching phenotype without having an obvious impact on macrochaetae. We highlight this tissue-specific sensitivity in our Discussion as described in our response to point #2 above. While we were able to use cellular reporter assays with Rbpj AOS variants and a DNA binding null mutant KRS to demonstrate titration of the Notch-coactivator (NICD) signal, to date we have been unsuccessful in using a similar approach to demonstrate titration of the co-repressor SHARP, which will require the design and testing of new reporter constructs that can better measure the gain and loss of repressor activity and is outside the scope of our current studies. Moreover, as there are multiple co-repressors in mammals that interact with Rbpj (Sharp, Kyot2, L3MBTL3, RITA), as opposed to a single co-repressor in *Drosophila*, interpreting the outcome of these types of experiments would be challenging.

4. *Why does Su(H)[lof]/+ suppress the Notch “nick” (loss-of-function) phenotype (Fig 3D)? In most previous studies, reducing the Su(H) dose has not modified the phenotype. Doesn't simple logic predict that, if anything, the Notch loss of function phenotype would be enhanced?*

We agree these results are non-intuitive regarding how lowering the Su(H) dose can suppress both Notch and Hairless phenotypes. We highlight this in our Discussion as described in our response to point #2 above.

5. *Why is there no effect on Cut, a target of Notch that is needed for wing margin? And also no affect an anterior E(spl)malpha? The story would be strengthened by extending the expression analysis to other genetic combinations, where the model predicts stronger defects.*

We focused on quantifying *E(spl)malpha-GFP* reporter expression over Cut levels because GFP could be analyzed without the need for using an antibody, whereas Cut requires primary/secondary antibodies which can amplify the signal and potentially make the signal non-linear. We focused on quantifying posterior wing margin cells over the macrochaetae because the *E(spl)malpha-GFP* enhancer is strongly activated by E-Box sequences that are bound by the Achaete/Scute proneural factors in macrochaetae. Hence, the *E(spl)malpha-GFP* enhancer is not as highly sensitive to changes in Notch strength within macrochaetae as compared to the wing margin cells. For example, see Castro et al (*Development* 2005, 132(15):3333-44. Doi: 10.1242/dev.01920) in which the authors found that expression of a *E(spl)malpha* reporter containing mutated Su(H) sites resulted in loss of expression in wing margin cells, while activity was maintained in macrochaetae due to strong proneural gene activation. We clarified this point in the legend to Figure 4.

6. *The genetics in the background papers are opaque but the authors need to be cautious in their explanation about the Su(H) “gain-of-function” alleles. The basis on which these alleles are gain of function is the enhancement of the phenotype from reduced Hairless. It’s important to note that the alleles are lethal over Su(H) deletions or null alleles and so might be considered loss of function at the same time. In this respect the observations are not 100% at odds with the mammalian data, although they do hint at a more complex explanation.*

We again apologize for this omission, which was also raised by Reviewer #1. Please see our response to Reviewer #1 – point 2 for how we addressed this issue.

7. *The sequence of the T4 and O5 mutants has been published in Flybase since 2010. The work here confirms the results. The fact that this is a confirmatory result and the data have been in the public domain for 11 years should be made clear.*

We agree that the sequences for the T4 and O5 alleles were previously reported in Flybase by Dr. Dolph and his group at Dartmouth. In fact, Drs. Gebelein and Kovall reached out to Dr. Dolph asking him if he and Emily Harding-Theobald would like to be authors on this paper, since it was originally their discovery. Because the personal communication on Flybase was not peer-reviewed and we included the original persons that communicated this finding as authors, we did not cite it. However, we have now clarified this point by adding the following statement to the Methods section:

*“Note, the sequencing of these Su(H) alleles were previously reported to Flybase via personal communication by Dr. Patrick Dolph^43^.”*

8. *There are other Su(H) alleles with similar properties, including Su(H)[S5], sequenced by Furukawa et al (1992). The R266H mutation has subsequently been shown to affect DNA binding. This fits with the current study, but they should acknowledge that at least one member of this class of Su(H) alleles was already known to affect DNA binding.*

We corrected this oversite and now included a more thorough description of the past studies using this allele. See our response to Reviewer #1, point 2 above.

9. *The images in Figure 4 are not very clear. At minimum a diagram with the positions of the macrochaetae would be helpful.*

Thank you for the suggestion. We added a diagram in Fig 4A’ to highlight the position of the macrochaetae as well as the wing margin cells.

Reviewer3:

1. *I recommend deleting the word "Notch" from the title. The described mutants do not act via "Notch Cofactor Titration ", they act via Su(H) cofactor titration, one of which is Notch.*

We made the recommended change.

2. *Avoid the use of phrases like “the presence of a single Su(H) allele” or “Drosophila with a single copy of the AOS-like Su(H) allele” (eg in the abstract and in the second paragraph of the Discussion). They do not clearly convey whether you are talking about the mutant allele heterozygous over the wildtype or over a deletion (the presence of a single mutant allele all on its own). Use the more common genetic terminology “heterozygosity for the Su(H) point mutant allele”, which implies over wildtype.*

We apologize for the confusion caused by our description of these alleles. We replaced “the presence of a single Su(H) allele” with “flies heterozygous for the Su(H) allele” and noted which allele we were using throughout the entire manuscript.

3. *A control is missing from the data of Fig. 7D. What are the levels of Tbp, Hey1 and Hes1 expression in the original MT line before CRISPR knockout of the RBPJ gene?*

We performed this control experiment in our previous paper Yuan et al Cell Reports (2019) “*Structural and Functional Studies of the RBPJ-SHARP Complex Reveal a Conserved Corepressor Binding Site*” in Fig. 3E, which shows that the Notch target genes Hes1 and Hey1, but not the Tbp control, are upregulated (de-repressed) in two independent CRISPR RBPJ knockout cell lines, but not in the control cells, i.e. no CRISPR knockout in the original MT cell line. We have modified the text describing Fig. 7D in order to make this point clearer.

4. *In Fig. 6F, the bottom panel should read "WB: a-Flag", not "IP: a-Flag". It would also help in this and the next two sub-figures (6G and 6H) to add the word "Input" where appropriate.*

We thank Reviewer3 for pointing out this error and making this suggestion to improve the legibility of this figure. We have made both changes in the revised manuscript.

5. *In Fig. 1C, please include the mouse mutant names in blue lettering, since they are encountered many times in the ensuing experiments.*

As suggested by Reviewer3, we have made these changes to Fig. 1C.

6. *With regard to Fig S3: Can cycloheximide block experiments be carried out for so long? Can cells survive without any protein synthesis for 3 days? Do we conclude that the half-life of Rbpj protein is >3 days?*

We agree with Reviewer3 that there are potentially technical issues with exposing cells to cycloheximide for 3 days; however, our grossly estimated half-life measurements of ~3 days for Rbpj is in line with what was determined for the half-life of Rbpj when it is deleted in vitro from mouse epidermal keratinocytes via Adeno-Cre treatment (t_1/2_ ~65 hrs) (Turkoz et al J. Invest. Dermatol, 2016). Moreover, we empirically determined the working concentration of CHX (0.5 µg/ml) for MK4 cells by examination of the morphological signs of toxicity prior to performing the CHX chase assay. While we did not explicitly measure ATP production, the coloration of phenol red in the media during the course of the CHX chase experiments suggested that the MK4 cells continue to perform cellular respiration, at least glycolysis and possibly oxidative phosphorylation, as acids (e.g., lactic acids or carbonic acids) continue to accumulate in the media. Despite these caveats, importantly our comparative studies suggest that there is no difference in the protein half-lives of Rbpj AOS variants compared to WT.

7. *In the Methods section: Is there a reason why the two EMSA experiments (purified proteins vs in vitro synthesized) were performed with a different target oligonucleotide?*

We apologize for the confusion, but the two EMSA experiments were performed in different labs, which use different oligonucleotide probes. However, both probes contain canonical RBPJ/Su(H) DNA binding sites, producing similar results.

8. *The qRT-PCR protocol is written twice in the Methods section. Delete one.*

We thank Reviewer3 for pointing out this error, which has been corrected in our revised manuscript.
